# *Staphylococcus aureus*, Including Meticillin-Resistant *Staphylococcus aureus*, among General Practitioners and Their Patients: A Cross-Sectional Study

**DOI:** 10.1371/journal.pone.0140045

**Published:** 2015-10-12

**Authors:** Barbara Michiels, Lien Appelen, Barbara Franck, Casper D. J. den Heijer, Stefaan Bartholomeeusen, Samuel Coenen

**Affiliations:** 1 Department of Primary and Interdisciplinary Care Antwerp—Centre for General Practice, Faculty of Medicine and Health Sciences, University of Antwerp, Antwerp, Belgium; 2 Faculty of Medicine and Health Sciences, University of Antwerp, Antwerp, Belgium; 3 Department of Medical Microbiology, Maastricht University Medical Centre/CAPHRI, Maastricht, the Netherlands; 4 Vaccine & Infectious Disease Institute (VAXINFECTIO)—Laboratory of Medical Microbiology, Faculty of Medicine and Health Sciences, University of Antwerp, Antwerp, Belgium; University Hospital Münster, GERMANY

## Abstract

**Background:**

The role of general practitioners (GPs) as reservoir and potential source for *Staphylococcus aureus* (SA) transmission is unknown. Our primary objective was to evaluate the prevalence of SA and community-acquired methicillin resistant SA (CA-MRSA) carrier status (including *spa* typing) among GPs and their patients in Belgium. The secondary objective was to determine the association between SA/CA-MRSA carriage in patients and their characteristics, SA carriage in GPs, GP and practice characteristics.

**Methods:**

The Belgian GPs, who swabbed their patients in the APRES study (which assessed the prevalence of SA nasal carriage in nine European countries; November 2010 –June 2011), were asked to swab themselves as well (May-June 2011). GPs and their patients had to complete a questionnaire on factors related to SA carriage and transmission. SA isolation including CA-MRSA and *spa* typing was performed on the swabs.

**Results:**

In eighteen practices 34 GPs swabbed patients of which 25 GPs provided personal swabs. The analysis was performed on 3008 patient records. Among GPs SA carriage (28%) was more prevalent than among their patients (19.2%), but CA-MRSA carriage was not present. SA was more prevalent among younger patients and those living with cattle. *Spa* typing SA and MRSA strains did not suggest correlation within practices or between patients and GPs, but chronic skin conditions of GPs and always handshaking patients by SA positive GPs were associated with more SA among patients, and hand washing after every patient contact with less SA among patients in practices with high antibiotic prescribing rates.

**Conclusion:**

No MRSA was found among GPs, although their SA carriership was higher compared to their patients’. *Spa* types did not cluster within practices, possibly due to difference in timing of swabbing. To minimise SA transmission to their patients GPs should consider taking appropriate care of their chronic skin diseases, antibiotic prescribing behaviour, handshaking and hand washing habits.

## Introduction

*Staphylococcus aureus* (SA) is a life threatening pathogen and a part of the commensal flora. Some SA clones are more virulent than others, although any SA genotype can become a pathogen under favourable host conditions[1]. Infections range from skin infections to severe pneumonia in vulnerable patients.

Some people are carriers of SA (most often found in the anterior nares) and their carrier status can be transient or more chronic for reasons not fully understood[2]. Colonisation of the anterior nares is considered as crucial in transmission and pathogenicity[2, 3].

*Meticillin-resistant Staphylococcus aureus* (MRSA) is no longer susceptible to meticillin and of great concern because of the high mortality following treatment failure. Overuse of antibiotics has been considered the most important cause, besides invasive procedures, immune and skin barrier deficiencies. Theoretically, there is no difference in transmission or infectious potency between SA and MRSA[4]. But, as MRSA occurs on more different body sites in persons with a weaker defence mechanism and a protective immune adaptation could occur among SA carriers, an higher infection potency is seen among MRSA carriers[5].

SA sources and transmission patterns have been examined extensively especially in hospital environments[6]. Recently, an interest has grown for *community-acquired MRSA* (CA-MRSA) and evidence for transmission patterns in the primary care healthcare setting is lacking.

The role of GPs as a reservoir and potential source for SA/CA-MRSA is unknown. GPs have short, frequent, and close contact with a diversity of vulnerable people. Risk factors for transmission are established for hospital healthcare workers (HCWs)[6]. Known risk factors for CA-MRSA carrier status are male gender, younger age, previous CA-MRSA acquisition, a hospital stay of long duration, working in the healthcare setting, chronic skin condition, chronic lung disease and antibiotic consumption[7]. Young children and pets in the household are important in SA/CA-MRSA transmission[8, 9]. Contact with cattle (cow, pig, chicken and horse) could increase the CA-MRSA carrier status[10].

This study is an add-on in one country (Belgium) to the APRES study (The appropriateness of prescribing antibiotics in primary health care in Europe with respect to antibiotic resistance-www.nivel.nl/en/apres). APRES aimed to establish the prevalence and antibiotic resistance of commensal SA, including MRSA, in healthy people in nine European countries, the differences between countries and the clustering of strain types within countries. The adjusted SA prevalence ranged from 12.1% (Hungary) to 29.4% (Sweden) and was 18.8% in Belgium among adults. In Belgium, the highest resistance rates (14.6% of the SA swabbed) were found for clindamycine[11]. The highest MRSA prevalence was recorded in Belgium (2.1% of all SA strains, 0.4% of all participants).

The primary objective of this add-on study was to evaluate the prevalence of SA and CA-MRSA carrier status (including *spa* typing) among GPs and their patients in Belgium. The secondary objective was to determine the association between SA/CA-MRSA carriage in patients and their characteristics, SA carriage in GPs, GP and practice characteristics.

## Materials and Methods

As mentioned before, this study build on the APRES study, which assessed the prevalence of SA nasal carriage among patients in nine European countries[11]. As an add-on, the GPs, who swabbed their patients in the APRES study, were asked to swab themselves as well. Only the Belgium participants were included.

In short, we recapitulate the methods used in the APRES study concerning the patients. Participating GPs were recruited from November 2010 to June 2011 in Belgium and asked to collect nasal swabs from 200 patients, aged 4 or older, who visited their practice for a non-infectious disorder. The recent use of antimicrobial drugs and hospital admission (previous 3 months), being an immunocompromised patient (eg. diabetes mellitus) or a nursing home resident were exclusion criteria.

Both anterior nares of the patient were swabbed with a charcoal swab from November, 2010 to June, 2011. The microbiological laboratory of the University Hospital of Antwerp isolated and identified SA. The isolated SA strains were then sent for antibiotic resistance testing to the microbiological laboratory of Maastricht University Centre, Netherlands. Real-time amplification of the *spa* locus with subsequent sequencing was performed on a random sample of 50% of the swabs (stratified by GP practice) including clustering of the *spa* types into *spa* clonal complexes (using the Ridom StaphType version 1.5 software package www.spaserver.ridom.de). More details are published elsewhere[11, 12].

Patients had to complete a questionnaire on factors which could possibly influence SA carriage such as having children younger than 5 years old in the family, keeping cattle (horses, cows, chickens or pigs) at home and chronic skin conditions (such as eczema, psoriasis, furunculosis) besides age and gender. Additionally, patients were asked about influenza and pneumococcal vaccinations, their frequency of GP consults in the last year and their work in care homes. The Belgian participating GPs were asked to swab themselves in both nares in the manner they applied to their patients from May to June, 2011. The microbiological analyses followed the same pathway as the patient swabs as described above. All GP SA positive swabs were *spa* typed.

GPs were also asked to complete the questionnaire, especially about their recent intake of antibiotics and hospitalisation (previous three months), their number of elderly care home visits and their hand washing and handshaking habits during the patient contacts.

Per practice, the antibiotic prescription rate (number of packages per 1000 patients) during the study period was obtained from the electronic health records by the Intego data collection system(Intego: integrated computerized network https://intego.be/nl/).

Ethical approval was obtained by the Medical Ethics Committee of the University Hospitals of KU Leuven and all patients and their GPs provided written informed consent before inclusion.

### Statistical analysis

Since patients and GPs were clustered in practices, and to account for possible clustering of patients’ SA (dependent variable) at GP practice level, multilevel logistic regression was performed. The model(s) included all relevant practice, GP and patient related co-variates (mentioned in Table 1) and significant interaction terms. Most variables were dichotomous (men or yes = 1; women or no = 0), except for age and antibiotic prescription rate which were continuous variables. The final model was defined by backward elimination of statistically insignificant interaction terms (a p-value higher than 0.01 was used as cut off, to avoid type I error due to multiple testing) using generalized estimating equations (SAS version 9.3).

**Table 1:** General characteristics of general practitioners (GPs) and patients, n (%).

	GPs n = 34	Patients n = 3008
**Men**	22 (64.7%)	1373 (45.9%)
**Age**	30–39y: 8 (23.5%)	55.4 y Range (4–107y)
	40–49y: 7 (20.6%)	
	50–59y: 11 (32.4%)	
	>60: 8 (23.5%)	
**Living in Belgium <3y**	NA	30/2963 (1.0%)
**Living with children ≤ 5y old**	9/29 (31.0%)	305/2980 (10.2%)
**Keeping cattle** ^a^	2/30 (6.7%)	45/3008 (1.5%)
**Number of GP visits / last year**	NA	No visits:105 (3.5%)
		1–4 visits 1481 (49.2%)
		>4 visits:1422 (47.3%)
**Working in hospital / nursing homes**	NA	139/3008 (4.6%)
**Resthome visits/month**	<10: 15 (50%)	NA
	10–19: 10 (33.3%)	
	>19: 5 (16.7%)	
**Working in children day care / nursery school**	NA	67/3008 (2.2%)
**Chronic skin condition**	4/29 (13.8%)	136/3008 (4.5%)
**Pneumococcal vaccine ever**	NA	492/2929 (16.8%)
**Influenza vaccination 2009/2010**	NA	1477/2996 (49.3%)
**Influenza vaccination 2010/2011**	NA	1368/2997 (45.7%)
**Antibiotic use (2011, Sep to Dec)**	5/30 (16.7%)	0^b^
**Hospital stay (2011, Sep to Dec)**	0	0^b^
**Handwashing before and / or after patient contact**	only after infection: 12 (41.4%) always: 17(58.6%)	NA
**Handshaking during patient contact**	12/29 (41.4%)	NA
**Antibiotic prescription rate / practice**	0.74 packages/1000 patients/day range 0.20–2.26)	NA

^a^ cattle: horses, cows, chickens or pigs

^b^ exclusion criterium

NA: not applicable

## Results

Of a total of 3132 Belgian patients, 3008 patient records were eligible for analysis, since 110 patients were excluded from the analysis because of missing or mismatch of swabs and background information and an extra 14 patients because they did not meet the age inclusion criterion (>4 years old).

Eighteen practices and 34 GPs swabbed patients. Twenty five of the GPs provided personal swabs. Table 1 shows the characteristics of the patients eligible for analysis and their GPs. The mean age of patients was 55,4 years; 32,4% of the GPs were aged between 50 and 59 years. More GPs than patients lived with young children (31,0% versus 10,2%), kept cattle at home (6,7% versus 1,5%) and suffered from chronic skin conditions (13,8% versus 4,5%).

Seven of the 25 swabbed GPs (28%) were SA positive, but no GP carried CA-MRSA. Of the swabbed patients 19,2% were SA carrier of which 2,1% were CA-MRSA positive (Table 2).

**Table 2:** Total number and percentage of SA and MRSA carrier-ship in patients and GPs.

**Patients** n = 3007	SA-positive carrier	576	19.2%
	of which MRSA pos	12	2.1%
	MRSA positive carrier	12	0.4%
**GPs** n = 25	SA-positive carrier	7	28%
	of which MRSA pos	0	0%
	MRSA positive carrier	0	0%

Of the 576 SA positive swabs of patients, 278 were *spa* typed which resulted in 138 different types (one was not typable). The most frequent encountered types were T002 (31%), t015 (13%), t021 (12%), t084 (10%), t091 (10%) and t166 (10%). There was no clustering of patient *spa* types within practices or a correlation between patient and GP *spa* types. The *spa* types of the 12 CA-MRSA positive patients were t008, t011(n = 2), t038, t062, t447(n = 3), t1923, t2346, t10847 of which only t008 and t447 were also found among non-resistant SA types (Table 3).

**Table 3:** SA/CA-MRSA-positive GPs and patients per practice with *spa* typing.

Practice	#GPs	#SA-pos GPs^a^	*Spa* type GPs	#Pat	#pat (pos SA /CA-MRSA)	#*spa* typed (SA/CA-MRSA)	*Spa* type (SA/CA-MRSA)
**1**	1	1	t8545	41	8/1	3/1	t002, t045, t166/t062
**2**	3	1	t316	227	50/1	24/1	3xt002, t005, t010, t012, 3xt015, 2xt021, 2xt084, t10501, t1084, t116, t11995, t166, t340, t393, t505, t6839, t864, t909/t447
**3**	1	0		114	15/0	7	t012, t11992, t127, 2xt230, t790, t7927
**4**	2	NS		199	46/1	22/1	3xt002, 2xt012,3xt015, t021, t050, t091, 2xt118, t121, t159, t1635, t166, t189, t 253, 2xt442, t6704/t447
**5**	1	0		226	45/3	21/3	3xt002, t015, t018, t021, t024, t056, t084, t091, t11994, t11996, t12000, t1248, t126, t148, t166, 2xt316, t493, t587/t2346, t1923, t011
**6**	6	3, 1xNS	t015, t091, t136	50	12/0	5	t015, t021, t026, t050, t571
**7**	3	0		155	18/1	8/1	t005, t084, t11999, t12001, t316, t359, t622, t790/t10847
**8**	2	0		198	29/0	14	3xt002, 3xt008, 2xt012, t021, t050, t084, t159, t267, t845
**9**	2	2xNS		258	48/1	24/1	3xt002, t005, t018, t081, t084, t085, 3xt091, t094, t12019, t136, 2xt159, t166, t186, t189, t209, t406, t561, t587, t867/t231
**10**	1	0		201	47/1	23/1	2xt005, 2xt015, t019, t021, t065, t078, t084, t091, t1046, t10600, t160, t166, t216, t4109, t437, t447, t535, t587, t6970, t8319, t949/t008
**11**	1	NS		111	12/0	6	t004, t12166, t233, t528, t7164, t772
**12**	1	NS		5	0	0	
**13**	1	0		229	38/0	19	4xt002, t019, t026, t065, t073, t091, t12014, t12016, t127, t1509, t2052, t209, t5081, t714, t726, t773
**14**	3	2, 1xNS	2xt645	237	50/1	24/1	4x002, t008, t012, t018, t019, t021, t084, t10042, t133, t166, t1875, t2066, t240, t267, t306, t352, t360, t491, t509, t589, t709/t038
**15**	1	0		192	37/2	17/2	2xt002, t015, t018, t021, t024, t026, t037, t091, t120, t12015, t12017, t136, t189, t279, t364, t559/t447, t011
**16**	2	0		246	58/0	29	5xt002, t015, t091, t095, t10982, 2xt1931, t12023, t1239, t1366, t196, t209, 3xt223, t2270, 2xt382, t548, t587, t6474, 2x662, t777, NT
**17**	1	NS		218	35/0	17	2x012, t015, t019, 3xt021, t065, t078, 2xt084, t10355, t12021, t12022, t166, t1996, t837
**18**	2	0		101	28/0	14	t008, t056, t091, 2xt136, t1451, 2xt166, t1712, t4679, t6804, t774, 2xt975
**Total**	**34**			**3008**	**576/12**	**277**	

^a^ No CA-MRSA in GPs

NS: not swabbed; NT: not *spa* typable

The multivariate model showed a positive association between the patient SA carriage and younger age and living with cattle as patient. Visiting their GP 1 to 4 times in the previous year seemed protective versus no visits, which was not the case for more than 4 visits.

SA carriage in patients was less likely when their GP was living with cattle, and more likely when their GP had a chronic skin condition, was SA positive and always handshaking during patient contacts, and in a practice with a low antibiotic prescribing rate, and an always hand washing GP. When GPs were always washing their hands before and/or after patient contacts, a high antibiotic prescribing rate was protective for patients. The opposite was true when GPs only washed their hands before and/or after infectious contacts(Table 4).

**Table 4:** Patient, general practitioner (GP) and practice factors associated with *Staphylococcus aureus (*SA) positive swabs in patients (multivariate model).

*Patient factors*	OR	95%CI
Sex (men (1) versus women (0))	1.05	0.93–1.18
**Age (older versus younger:difference of 10 years)**	**0.85**	**0.80–0.90** ^**b**^
Living with children less than 5 years old^a^	1.32	0.93–1.85
Working in hospital / nursing homes^a^	0.78	0.41–1.47
Working in crèche / nursery school^a^	1.34	0.76–2.36
**Living with cattle**	**2.17**	**1.34–3.52** ^**c**^
Number of GP visits/year		
**1–4 versus 0 (ref category)**	**0.50**	**0.33–0.74** ^**d**^
>4 versus 0	1.07	0.87–1.32
Chronic skin condition^a^	0.85	0.46–1.58
Pneumococcal vaccine ever^a^	0.73	0.45–1.18
Influenza vaccination 2009/2010^a^	1.19	0.71–2.02
Influenza vaccination 2010/2011^a^	1.00	0.64–1.59
***GP and practice factors***		
Age (older versus younger:difference of 10 years)	1.41	0.69–2.90
Living with children less than 5 year old^a^	1.20	0.97–1.50
**Living with cattle** ^a^	**0.67**	**0.59–0.76** ^**b**^
**Chronic skin condition** ^a^	**1.40**	**1.20–1.63** ^**b**^
Number of eldery living home visits/month <10 times (ref category)		
10–19 times	1.19	0.97–1.46
>19 times	1.03	0.82–1.30
**GP-SA pos versus neg and always handshaking during patient contact** ^a^	**1.97**	**1.51–2.57** ^**b**^
GP-SA pos versus neg and never handshaking during patient contact	1.10	0.83–1.47
**Handshaking always versus never during patient contact when GP is SA pos** ^a^	**1.61**	**1.21–2.14** ^**e**^
Handshaking always versus never during patient contact when GP is SA neg	0.90	0.74–1.09
**Hand washing always versus only after infection + AB_prescribing = 0.5**	**1.92**	**1.57–2.35** ^**b**^
Hand washing always versus only after infection + AB_prescribing = 2	0.84	0.62–1.15
**AB_prescribing = 2 versus 1 + hand washing only after infection** ^a^	**1.21**	**1.13–1.30** ^**b**^
**AB_prescribing = 2 versus 1 + hand washing always**	**0.80**	**0.72–0.89** ^**b**^

^a^ yes = 1 versus no = 0

^b^ p < 0.0001

^c^ p = 0.002

^d^ p = 0.0006

^e^ p = 0.001

## Discussion

Never before a link was examined between SA carriage of patients and their GPs. This APRES add-on study revealed that among GPs SA carriage (28%) was more prevalent than among their patients (19.2%), but without carrying CA-MRSA.

The prevalence of CA-MRSA carrier status in the community is generally low: 0%-2% in the general population whereas the SA prevalence is higher: 20%-27%[9, 11, 13, 14]. The Belgium patients in the APRES study with a carrier status of 19,2% are situated at the lower end.

A review calculated that 23,7% of 10589 HCWs (working mainly in hospitals) carried SA and 4,6% of 33318 HCWs carried MRSA[6]. A study in the Netherlands (2006) among 395 GPs attending a conference revealed that 33% were SA carriers and no one carried MRSA. The authors suggested that patients were most probably infecting GPs and not the other way around, but this statement is contra intuitive and not proven[14]. A small study in the West of Ireland (2005) showed an incidence of 7,7% CA-MRSA among 78 GPs, but SA carrier status was not described[15]. These results indicate that depending on local circumstances a large difference in MRSA incidence can be seen. The results of our study (28% SA carriers in Belgian/Flemish GPs with 0% CA-MRSA) resembles more the Dutch findings.

As reported previously there is a low prevalence of CA-MRSA in healthy people in several countries[11] and surprisingly also among GPs. The review of Albrich et al showed that MRSA was lower among HCWs in an ambulatory setting compared to an hospital setting (3,4% versus 5,4%)[6]. CA-MRSA are found on other body sites than the anterior nares, which was not tested in our study[5, 8]. Known factors which influence carrier status in HCWs in hospital settings are not fully applicable to GPs[6].

Factors determining SA/CA-MRSA carrier status in healthy people are not yet fully understood, including those factors that influence intermittent versus persistent carrier status. Our study confirmed that younger people and those living with cattle are more prone to SA carriage. The protective role of visiting their GPs 1 to 4 times in the previous year compared to no visits is not easily explained with the information collected in our study. We could not show that living with young children, working in hospitals or nursing homes, or working in a children day care centre or nursery school was of significant influence.

Although we know that carrier status in the anterior nares is a source of transmission and infection, GPs can play a direct (by being a carrier) or indirect role (without being a carrier) in transmission to patients, which is also described in hospitals[6]. Although we had to deal with a small GP sample size, we could show that GP characteristics such as chronic skin conditions were associated with patient carrier status[6, 8]. We could confirm a decrease in SA colonisation with age[16]. Only one GP cohabited with cattle and was SA negative which might explain the unexpected reverse relationship. There was no proven influence of GPs with young children. We also showed that handshaking and hand washing habits could be important promoting and/or protecting factors. Besides the anterior nares the hands are a second important site for SA/(CA-)MRSA transient and persistent carriage[6], which underlines the importance of appropriate hand washing habits. The number of antibiotics prescribed in the GP practice was an interfering factor as could be expected. Other unknown confounding could alter the associatons found in our study between GP characteristics and patient carrier status such as obesity, smoking habits[17], the use of nasally applicated decongestives/antiseptics, the use of gloves by the GPs, contact and cleaning frequency of contaminated surfaces in GP practices and the frequency of close contact to other patients and practice assistant personel. The difference between intermittent and persistent carrier status could not be made in our study. Davies et al estimated that the duration of colonisation with CA-MRSA in patients could last from 2 weeks to 9 months and that 20% of those colonised patients are persistent carriers[8]. Persistent carriage is related to chronic skin diseases or chronic upper respiratory tract infections such as sinusitis[6].

The *spa* types revealed a broad range of types with a few more prevalent ones, but without any straightforward clustering within practices or any relationship between GPs and patients. This was possibly due to the collection of swabs in patients spread over an eight month period, the collection of swabs in GPs only in the last two months of this period, i.e. later than in most of their patients, and our GP sample perhaps being too small. In addition, the clustering of *spa* types in *spa* complexes was of no additional use to describe transmission direction in our study[18]. The heterogeneity among SA types was also seen in other European countries[11] and in other settings[16, 19]. Also the relationship between hospital types and community types is diverse. We know that *spa* types are evolving over time in the same person, which makes the interpretation even more complex[4, 19, 20]. We have to keep in mind that *spa* types are not predicting virulence or endotoxin production, which are connected to other genome regions[21].

Some limitations of this study are to be mentioned. First of all the small GP sample size, which prohibited the evaluation of factors of influence on GP carrier status. There was also a difference in the timing of swabbing between most patients and the GPs, long enough to allow SA *spa* types to evolve so that transmission patterns between GP and patients could be hidden. Despite this shortcoming, transmission between GPs and patients and association between GP characterisitics and patient carrier status are still plausible, but need further confirmation. Missing data in swabs and in questionnaire answers made the multivariate analysis less robust, but despite this drawback we still revealed relevant findings with low p-values (Table 4). By nasal swabbing only once in this cross sectional study, we probably missed some carriers and could not distinguish between intermittent and persistent carriage, neither indicate the direction of transmission. We can only speak of association with possible risk factors and not of causality.

In conclusion, among Belgian GPs, positive SA carriers were more prevalent than among their patients, although no GP carried MRSA. *Spa* typing could not underscore transmission patterns and direction between GPs and patients, which is to be considered a limitation. A larger longitudinal cross sectional study, swabbing patients and GPs at the same time at several time points and taking into account other possible confounders is warranted to learn more about the exact role of GPs in SA/CA-MRSA transmission in particular and transmission of infectious pathogens in general. Some of the many factors, which influence the SA/MRSA epidemiology, GPs can handle, for example appropriate care for chronic skin diseases, antibiotic prescribing behaviour, handshaking and hand washing habits.
